# Toward a global health approach: lessons from the HIV and Ebola epidemics

**DOI:** 10.1186/s12992-018-0435-9

**Published:** 2018-11-22

**Authors:** Gilles Raguin, Pierre-Marie Girard

**Affiliations:** 1 Institute of Applied Medicine and Epidemiology (IMEA), Fondation Internationale Léon Mba, 46 rue Henri Huchard, 75018 Paris, France; 2 Department of Infectious and Tropical Diseases & Inserm, UMR S 1136 and UPMC, University of Paris 6, St Antoine Hospital, APHP, IMEA Fondation Internationale Léon Mba, Paris, France

**Keywords:** HIV, Ebola, Global health

## Abstract

**Background:**

The imposing burden of non-communicable diseases, emerging infectious diseases, climate change, environmental consequences, migrations, urbanization, and other challenges, faced in a context that strives to make universal health coverage (UHC) a reality, compels global health professionals to ask: how do we construct a “global” roadmap that is both realistic and effective?

To move forward and begin to answer this question, we draw on lessons and experiences gained during the “global” health crises triggered by the HIV and Ebola pandemics.

**Main text:**

Improving the early response and committing to the long haul; developing inter-disciplinary and inter-sectoral responses; designing comprehensive and versatile interventions; and, most importantly, to work closely and effectively with civil society and communities are some of the critical elements that were identified.

The health sector has changed dramatically in recent years; new tools and innovative technologies are transforming the culture and practice of public health. This calls for a new vision.

Reprioritizing primary health care and community engagement, repositioning approaches to meet people’s needs, applying integrated disease management to respond to problems caused by the silo approach, implementing UHC, and ensuring equity are some of the new strategies.

**Conclusion:**

These strategies must all undergo a mandatory revolution in health governance—locally and globally. It should be obvious that nothing can be improved on a global or sustainable scale without re-examining the architecture and governance of major funding and international organizations dedicated to health.

Pressing economic, demographic, and climate issues related to health underscore the urgent need for these changes.

## Background

100 years after its outbreak at the start of the twentieth century in Central Africa [1], the HIV epidemic has not ended. Although we have seen positive indicators from the African continent, many problems continue to hamper controlling this pandemic.

New health challenges are appearing on the global health landscape. The Ebola virus disease (EVD) outbreak, a cataclysmic health crisis, revealed the desperate state of health systems in low-resource countries. Chronic noncommunicable diseases—diabetes, cancer, and cardiovascular diseases—kill more people today than infectious diseases, even in Africa. Meanwhile, infectious diseases, such as Zika, plague, and yellow fever, continue to emerge or re-emerge. Climate change and food security are serious concerns. And we must not forget previous scourges lurking behind the scenes, better controlled today but still very present: tuberculosis, malaria, and infant and maternal mortality.

In the face of this imposing burden, in the context of UHC promised by the new United Nations Sustainable Development Goals [2], the key question confronting global health professionals is: how do we construct a “global” roadmap to health and health equity that is both realistic and effective?

Global health, in effect, no longer prioritizes a disease, a specific aspect of the health system, or one continent over another, but instead focuses on improving health and health equity for everyone on the planet. A huge endeavor, indeed!

To move forward, let us draw on lessons and experiences we have personally gained during the “global” health crises triggered by the HIV and Ebola pandemics.

## Main text

### Response must be early, appropriate, and sustainable

Unfortunately, under-estimating needs at the start of epidemics and over-estimating the resulting outcomes once the epidemic ends are consistent observations. Nevertheless, everything is there: identifying the crisis early on; quickly rolling out efforts and resources, without being swayed by panic or denial; and committing to the long haul.

The HIV epidemic, tracing back to the early twentieth century followed by its “discovery” in the 1980s, was largely under-estimated by officials at the time, by their own admission, resulting in the loss of precious time for an international response. Even today, the pandemic continues to gain ground despite slowing down or even reversing in some countries. The danger lies in celebrating too soon while letting down one’s guard. For this reason, announcements of its eradication by 2030 are counter-productive: it will presumably take several more decades of effort before this pandemic is truly brought under control, especially with the current worrisome emergence of antiretroviral resistance.

Similarly, there is broad consensus that the local and international response to the EVD epidemic of 2013–2016 was both late and poorly adapted. A constellation of factors—ignorance of or under-estimating early warnings, the inertia of responsible institutions, and unsuitability of the initial responses—allowed the epidemic to reach a scale unequaled in the history of this disease. And despite promises, too little funding is now mobilized on the ground to prevent the onset of other epidemic episodes, even as the animal reservoir, which remains poorly understood, can be re-activated at any moment. Health systems strengthening, in terms of infection prevention and control, remains in its nascent stages in most African countries, despite this serious warning.

Acting early and appropriately also means being able to mobilize the necessary human and financial resources, which generally exceed what low-resource countries are able to raise. The fight against AIDS lacked financial support for a long time until the Global Fund to Fight AIDS, Tuberculosis, and Malaria was created in 2002, followed by the US President’s Emergency Plan for AIDS Relief (PEPFAR) in 2003. The recent positive trends in the fight against AIDS in Africa are, mostly, a direct consequence of these investments. Similarly, with Ebola, funding was mobilized too slowly, partially explaining the delays in combatting the epidemic. State and international NGO resources were quickly depleted, leaving the international community to mobilize emergency funds solely through unstructured channels. Nevertheless, the lesson bore fruit with the creation, albeit late and rudimentary, of the WHO and World Bank emergency funds. However, the good news is that this epidemic has been controlled, with time and sustained efforts.

### Global, inter-disciplinary, and inter-sectoral responses

The fight against HIV has shown that the biomedical response is only one aspect, necessary but insufficient, of an effective response to pandemics. The essential role that the humanities and social sciences play in information, reduction of fear and stigma, prevention, screening, treatment adherence, and control policies is well known [3, 4]. There can be no effective response if other sectors of society are not also mobilized: financing, education, media and communication, security forces, and diplomacy. An effective public health approach is political and engages all levels of public power and society.

Because of the errors made at its beginning, the 2013–2016 EVD epidemic clearly showed how much the combination of disciplines and sectoral interventions finally brought the pandemic under control. Communities and care givers paid dearly, in human lives, for the initial lack of collaboration between biomedical actors, security forces, anthropologists, crisis communication specialists, animal specialists, politicians, and diplomats. Rejection of sanitary measures imposed on populations with no explanation or consultation, abuse of power and imprisonment, abusive border closures, riots, and murdered health care staff: these are some of the events that could have been avoided or minimized by combining the expertise and interventions of all relevant actors. The development of a “One World, One Health” approach, which specifically aims to unify animal and human health actors around a single, common, “global” goal is an excellent initiative, though still in its inception.

The response must be comprehensive and versatile on day-one, combining prevention and treatment, delivery of hospital care and primary health care, community participation and international mobilization, grassroots outreach with communities as well as sophisticated interventions. International humanitarian interventions—disparate, poorly coordinated, and even competing against each other—often factor into the disorganized and inappropriate management of health emergencies by States. Westerners often apply a technologically sophisticated, and sometimes dogmatic, approach that is poorly adapted to local contexts and can even be counter-productive when it results in marginalizing the essential role of communities. All components of the health system must be engaged and strengthened. The approach must be global and coordinated while based on involving local actors [5]. Civil society should always be a key stakeholder in interventions, starting with their design.

The health sector has changed dramatically in recent years, and this calls for a new vision.

Information and communication is one area where innovations offer considerable improvements: rapid and relevant information for public health actors and decision makers, enabling them to make decisions quickly and early; and rapid and intelligible information for populations, care givers, and actors overseeing health and civil security, allowing them to better prevent the spread of infections and their consequences. New technologies enable this, whether at the local level with tools to enhance connectivity, or at the regional and even global level, with big data, spatial data integration, and social and scientific networks [6]. Their potential is considerable, as are the related issues: ownership, data management, transparency and verification of information, and political issues. These new tools will transform the culture and practice of public health.

The supply side of health care—how we provide health services closest to those needing them that are best tailored to their needs—will also see major changes. Beyond waking international actors up to reprioritizing primary health care and community engagement, the essential nature of which was rediscovered during the Ebola crisis, major changes have begun. These include repositioning approaches toward people’s needs, task shifting and sharing to mitigate serious health workforce deficits across all continents, integrated disease management to respond to problems caused by the silo approach, UHC and equitable access to quality care and affordable health commodities.

All these must undergo a mandatory revolution in health governance—locally and globally. As with global warming, it should be obvious that nothing can be improved on a global or sustainable scale without re-examining the architecture and governance of major funding and international organizations dedicated to health, which are too vertical (devoted specifically to a single disease or group of diseases), poorly coordinated, and insufficiently integrated. In this global framework, an approach based on the concept of global public goods seems the most appropriate. Civil society is increasingly, and quite justly, demanding more participatory governance, both locally and globally. Lastly, in an interconnected world, transnational networks, partnerships, and coalitions—especially between international and local NGOs—as well as economic clubs and networks are rapidly developing and will transform the world of health.

Here, politicians have an immense responsibility. These rapid changes must indeed be accompanied by a revision of legal, ethical, and societal rules and frameworks (UHC, the right to health, participatory governance, climate and environment, health determinants, etc.). Delivery of health care services must be reconfigured so the interlocking components—care giver status and training, the health system, social justice, transparency, and accountability—run efficiently [7]. The nature, scope, and extent of international conventions, agreements, and regulations (including international health regulations) must be updated. These changes are urgent as economic, demographic, and climate issues related to health become even more pressing.

## Conclusion

The key question confronting global health professionals today is: how do we construct a “global” roadmap towards health and health equity that is both realistic and effective?

Drawing on lessons and experiences gained during the “global” health crises triggered by the HIV and Ebola pandemics provides some insight and brings to the conclusion that nothing can be improved on a global or sustainable scale without a revolution in health governance—locally and globally.
